# Small intestinal bacterial overgrowth in variant transthyretin amyloidosis (A-ATTRv)

**DOI:** 10.1186/s13023-025-03727-y

**Published:** 2025-05-02

**Authors:** Ana Moreno García, Mireia Grimalt Oliver, Juan González-Moreno, María Antonia Ribot-Sansó, Eugenia Cisneros-Barroso, Inés Losada López

**Affiliations:** 1 Universitat de les Illes Balears, Palma, Spain; 2 Endocrinology and Nutrition, Hospital Universitario Son Llàtzer, Crta Manacor Km 4, Balearic Islands, Palma, 07198 Spain; 3 Sudden Death and TTR Amyloidosis, Balearic Research Group in Genetic Cardiopathies, Health Research Institute of the Balearic Islands (IdISBa), Balearic Islands, Palma, Spain; 4 Internal Medicine Department, Hospital Universitario Son Llàtzer, Crta Manacor Km 4, Balearic Islands, Palma, 07198 Spain

**Keywords:** ATTR, Transthyretin amyloidosis, Small intestine bacterial overgrowth, Digestive symptoms, Breath test

## Abstract

Variant transthyretin amyloidosis (A-ATTRv) can lead to sensory, motor, and autonomic neuropathy, as well as a variety of gastrointestinal (GI) disorders. While previous studies have explored gastrointestinal symptoms in A-ATTRv, no studies have definitively examined the role of bacterial overgrowth, such as SIBO, in exacerbating these symptoms. Identifying the presence of SIBO in A-ATTRv patients could lead to better-targeted treatments for gastrointestinal complications.

We conducted a cross-sectional, unicentric observational pilot study, analysing the presence of SIBO using a lactitol breath test in 39 individuals carrying the V30M mutation: 21 asymptomatic carriers and 18 patients with A-ATTRv. We did not find a higher prevalence of SIBO, among patients with A-ATTRv compared to asymptomatic carriers. Though no significant relationship between SIBO and A-ATTRv was found, notable differences in gastrointestinal symptoms suggest these may be independent of SIBO. Furthermore, no relationship was found between the presence of SIBO and gender.

Given the limitations of this pilot study, we did not find a relationship between A-ATTRv and intestinal microbiota disorders. Future research with larger sample sizes and more sensitive diagnostic tools is required to further explore this potential link.

## Introduction

### Transthyretin amyloidosis

Variant transthyretin amyloidosis (A-ATTRv) affects approximately 10,000 people worldwide. The V30M variant represents 73% of all A-ATTRv cases [1], with higher prevalence in Portugal, Brazil, Japan, Sweden, and Spain. In Spain, there is no national registry for A-ATTRv cases, but it is estimated that there are over 350 cases [2]. Mallorca is the largest focus in Spain and A-ATTRv is considered endemic in this area.

In A-ATTRv patients, amyloid fibril deposits cause systemic symptoms that can lead to serious complications within 10–15 years [3, 4]. It is predominantly associated with sensory, motor, and autonomic neuropathy [5, 6]. Additionally, there can be other symptoms such as cardiac, gastrointestinal (GI), ocular, renal, central nervous system disorders, and carpal tunnel syndrome [5, 7].

The frequency of GI complications varies among different populations and variants [3, 8]. However, in A-ATTRv, it has been shown that almost all untreated patients develop these alterations over time [9]. The pathogenesis of GI manifestations is not fully understood, but most symptoms are generally suggested to arise from GI tract motility disorders caused by autonomic neuropathy [10]. Additionally, depletion of enteric nerves [11, 12] and endocrine cells [13], along with a reduction in interstitial cells of Cajal [14], have been observed. This can lead to decreased GI tract motility and gastroparesis, frequently seen in A-ATTRv [15, 16].

These alterations result in impaired GI tract function, significantly impacting patient quality of life [17]. Upper GI tract symptoms include early satiety, nausea, and vomiting, while constipation, alternating constipation and diarrhea, and persistent diarrhea are symptoms from the lower GI tract [18]. The early onset of GI disorders, including diarrhea and malnutrition, negatively influences the survival of these patients [19, 20].

Due to the impairment in GI motility shown in A-ATTRv, there could be a reduction in the body’s ability to clear bacteria from the small intestine, thus increasing the risk of bacterial overgrowth.

### Small intestinal bacterial overgrowth (SIBO)

SIBO is defined as a clinical syndrome characterized by GI symptoms resulting from an excessive number of bacteria in the small intestine.

The human gut microbiota represents a huge ecosystem of microorganisms that has implications in different mechanisms, such as motility and integrity of the small intestine, gastric and pancreatobiliary secretions and local immunity. SIBO emerges as a dysbiosis resulting from the disruption of this environment and is defined by an increased bacterial load in the small intestine. In healthy individuals, the usual bacterial levels are around < 10^4^ colony-forming units per milliliter (CFU/mL), with counts above 10^5^ CFU/mL described as SIBO [21, 22].

The clinical presentation of SIBO is nonspecific and characterized by many digestive symptoms. This makes a challenge for proper diagnosis and complicates the accurate estimation of its real prevalence. Currently, the prevalence of SIBO in the general population is unknown. Different studies have documented rates from 2.5 to 22%, with the increase in prevalence with age and comorbidities [23–25]. Discrepancies in prevalence can be attributed to the fact that SIBO can be asymptomatic or have different symptoms such as abdominal bloating, pain or altered bowel habits, which could be also caused by other conditions [26].

Disruption of GI homeostasis can predispose patients to develop SIBO, by different mechanisms [25]: intestinal dysmotility, alteration of GI secretions, anatomical abnormalities and impaired intestinal immunity.

#### SIBO subtypes

Currently, four types of SIBO are distinguished, considering the main symptoms experienced by patients and the excess final gas product.

- Hydrogen SIBO (H_2_ SIBO): Commonly manifests with weight loss, abdominal distension, flatulence, diarrhea, pain, and bloating. The frequency and severity of these symptoms can vary among patients [27]. Vitamin B12 deficiency can also be a consequence, as bacteria use this vitamin competing with normal absorption [28].
- Methane SIBO (CH_4_ SIBO): Classic symptoms include constipation and irritable bowel syndrome, and it can manifest in both large and small intestine [29]. Other symptoms include bloating, abdominal pain, and decreased intestinal motility, which can lead to confusion with H_2_ SIBO.
- Hydrogen Sulphide SIBO (H_2_S SIBO): The symptoms include constipation or diarrhea, as well as a very strong smell flatulence caused by hydrogen sulphide, a very toxic, foul-smelling, and inflammatory gas. It is usually accompanied by extra-digestive symptoms such as headaches, fatigue, acne and inflamed gums [30].
- Small Intestinal Fungal Overgrowth (SIFO): It refers to overgrowth of fungi, usually *Candida spp*. or *Aspergillus spp*. Its diagnosis is complicated because the main symptoms are very similar to bacterial overgrowth. It presents a real threat, especially affecting immunocompromised individuals. However, this condition has also been observed in healthy individuals, although the reasons are still unknown [31].

Currently, breath tests are used as a safe and non-invasive technique for diagnosing H_2_, CH_4_ and H_2_S SIBO [30, 32]. These tests measure the concentration of these gases in exhaled air after the oral administration of either lactulose or glucose. Although they are accessible and cost-effective, they have a limited sensitivity and specificity. A breath test using these substrates can have a sensitivity between 42% and 54.5% and a specificity between 70.6% and 83.2% [33]. The diagnostic concordance between small intestine aspirate culture and the breath test is approximately 65% [29]. It should be noted that this test can give false positive results, as lactulose can increase intestinal motility and reflect colonic rather than small intestine fermentation [34].

Abnormal small bowel motility is a common feature of ATTRv amyloidosis and may predispose patients to conditions such as SIBO. Wixner et al. [35]. demonstrated that ATTRv patients exhibit significantly more motility abnormalities compared to healthy controls. In addition, alterations in gut microbiota composition have been observed in ATTRv patients, as reported by Chen et al. [36]. These disruptions in motility and microbiota balance likely contribute to the development of gastrointestinal symptoms and SIBO. Given the high prevalence of GI symptoms in patients with A-ATTR-V30M and the established link between GI dysmotility and SIBO, our study aims to investigate whether SIBO is more prevalent in A-ATTRv patients compared to healthy variant carriers.

## Materials and methods

To verify the hypothesis, a pilot project was proposed to establish the frequency of SIBO in patients with A-ATTRv from a cohort of the Son Llàtzer University Hospital. This general objective was enlarged with the following specific objectives:

- Analyse the presence of SIBO with the lactitol breath test in carriers of pathogenic TTR variants without a diagnosis of A-ATTRv and patients with A-ATTRv.
- Compare the frequency of different types of SIBO in asymptomatic carriers of pathogenic TTR variants and patients with A-ATTR-v.
- Analyse the relationship between SIBO and gender.
- Describe GI symptoms in patients with SIBO.

### Study design

We designed a cross-sectional, unicentric observational pilot study, at Son Llàtzer University Hospital, with participants selected between November 2023 and December 2023.

### Inclusion and exclusion criteria

Two groups of individuals were included:

1. Patients with A-ATTRv V30M.
2. Individuals carrying the V30M variant without an A-ATTRv diagnosis.

The individuals included in this study are part of a cohort undergoing regular follow-up at the multidisciplinary A-ATTR unit of Son Llàtzer University Hospital. The diagnosis of A-ATTRv was established according to the hospital’s protocols. All subjects underwent genetic testing for TTR Carriers were defined as individuals with a pathogenic TTR gene variant who exhibited no symptoms of polyneuropathy (PNP) and had normal results on small fiber/autonomic function tests and electroneurography.

Patients were classified as those meeting the diagnostic criteria for PNP at our hospital, which required the presence of symptoms consistent with sensorimotor polyneuropathy or small fiber/autonomic neuropathy, along with either abnormal electroneurographic findings or at least three positive tests for small fiber/dysautonomic dysfunction (Sudoscan®, QST, R-R interval variability, and/or SSR).

All patients underwent standardized nerve conduction studies as part of a structured follow-up protocol. Our neurophysiological assessments include bilateral sensory nerve action potentials (SNAP) and sensory conduction velocities (SCV) for the sural nerve, superficial peroneal nerves, and the median and ulnar nerves at the second and fifth fingers. Additionally, we evaluate compound motor action potentials (cMAP) and motor conduction velocities (MCV) for the bilateral distal common peroneal nerve, posterior tibial nerve, and the median and ulnar nerves in the dominant hand.

In cases of suspected carpal tunnel syndrome (CTS), the contralateral side is also assessed. These evaluations include distal motor latencies (DML), conduction velocity, and the identification of conduction blocks, ensuring a comprehensive analysis of nerve function. All nerve conduction studies are interpreted using standardized reference values [37].

To diagnose A-ATTRv with cardiac involvement, the presence of a pathogenic TTR gene variant along with Perugini grade 2–3 uptake on cardiac scintigraphy marked with pyrophosphates, excluding light chains, is required. In both, ATTRv with polyneuropathy and cardiomyopathy, a pathogenic variant in the transthyretin gen is obligate. Digestive symptoms, defined as diarrhea, constipation, early satiety, or flatulence, were analysed.

The exclusion criteria were being under 18 years old, being pregnant, the use of antibiotics in the last month, liver transplant or not signing the informed consent.

### Participant selection

Initially, a review and selection of patients meeting the inclusion criteria were carried out, excluding those with any pre-established exclusion criteria. Prior to the study, all participants were informed in detail, both in writing and verbally, about the study’s development. They were instructed on the objectives, methodology, and guidelines to follow during the lactitol breath test for SIBO prevalence analysis. After reviewing the patient information sheet and verifying that the subject met all inclusion criteria and none of the exclusion criteria, the informed consent document was signed. The study was conducted according to Good Clinical Practice standards and the International Conference on Harmonization Guidelines. Data were anonymized and handled confidentially according to current data protection legislation.

### SIBO test

All participants underwent a H_2_ and CH_4_ breath test to diagnose these two subtypes of SIBO. Gas samples were collected at 30-minute intervals over a 210-minute period following the administration of 10 g of lactitol. The exhaled concentrations of H_2_ and CH_4_ were analyzed using the Quintron BreathTracker SC, which employs gas chromatography with a CO_2_ dilution correction factor to ensure precise and accurate measurement of intestinal fermentation gases. Probiotics and proton pump inhibitors were discontinued 15 days prior to the test, and participants were instructed to abstain from alcohol and dietary fiber for 24 h beforehand. The breath test was conducted after a 12-hour fasting period, during which participants refrained from exercise and smoking [34]. It is important to note that H_2_ and CH_4_ breath tests can yield discordant results due to variations in gas production by intestinal bacteria, differences in transit speed, and other dietary and lifestyle factors. Therefore, simultaneous measurement of H_2_ and CH_4_ can enhance the accuracy of the test [29]. Also, carbon dioxide (CO_2_) values were used to assess the quality of the samples, with values below 1.7% indicating inadequate sample integrity, making H_2_ and CH_4_ values unacceptable [29, 38].

The diagnostic criteria used in this study were based on established guidelines from the American Gastroenterological Association and the North American Consensus. According to the American Gastroenterological Association, SIBO was confirmed when H_2_ levels of 20 parts per million (ppm) or higher appear within the first 90 min compared to the baseline value. Similarly, IMO was confirmed when CH_4_ levels of 10 ppm or higher are found at any point in the curve. These criteria are supported by the North American Consensus, which also recommends a rise in hydrogen of ≥ 20 ppm from baseline within 90 min during glucose or lactulose breath testing as diagnostic of SIBO, and methane levels ≥ 10 ppm at any point as indicative of methanogen colonization [39, 40].

### Sample size

SIBO prevalence in the healthy population can vary based on studies and demographic characteristics. However, it is estimated that approximately 2.5–22% of the general population may present SIBO [23–25]. It is important to note that prevalence can be different depending on geographic region, age, and other factors. Given the exploratory nature of this study and the limited cohort of patients at our centre, a feasibility-based sample size was determined. This study aims to establish the frequency of SIBO in this population.

### Statistical analysis

Data analysis was performed using IBM® SPSS® Statistics v23.0 software. The significance level was set at α = 0.05. After confirming data normality, continuous variables were expressed as mean and standard deviation. Numbers and percentages were used for categorical variables. The comparison of categorical variables was performed using Fisher’s exact test, and non-categorical variables using the T-test.

## Results

A total of 39 individuals carrying the V30M mutation were included in the study, being 21 asymptomatic carriers and 18 patients with A-ATTRv V30M. Within the group of individuals with A-ATTRv, 3 patients had exclusively cardiac complications, 5 presented advanced-stage neurological symptoms, and the other 5 patients displayed early-stage neurological symptoms. The remaining 5 patients exhibited mixed involvement, characterized by the coexistence of both polyneuropathy and cardiomyopathy.

The presence of SIBO was evaluated in the 39 selected individuals using the lactitol breath test, as detailed in the materials and methods section. Only two subjects were excluded due to inconclusive breath test results. Therefore, the final analysis included 37 individuals, of whom 20 were carriers and 17 had A-ATTRv, with the characteristics shown in Table 1.

**Table 1:** Characteristics and digestive symptoms of the study population

	Total (*n* = 37)	Carriers (*n* = 20)	ATTRv patients (*n* = 17)
Age (x̅) (range)	55.3 (24–86)	48.9 (24–74)	62.8 (32–93)
Age of diagnosis (x̅)	-	-	55.4
Gender (Female)	21 (56.8%)	12 (60%)	9 (53%)
GI Symptoms	15 (40.5%)	2 (10%)	13 (76.5%)
Diarrhea	10 (27.0%)	1 (5%)	9 (52.9%)
Diarrhea and constipation	9 (24.3%)	2 (10%)	7 (41.2%)
Abdominal distension	6 (16.2%)	1 (5%)	5 (29.4%)
Early satiety	4 (10.8%)	0 (0%)	4 (23.5%)
Flatulence	6 (16.2%)	2 (10%)	4 (23.5%)
None	22 (59.4%)	18 (90%)	4 (23.5%)

Statistical significance was evaluated using Fisher’s exact test (*p* < 0.0001)

In the study population, no significant differences in sex were found between the groups of carriers and patients with A-ATTRv (60% vs. 53%, *p* = 0.7463). There was a statistically significant difference in the prevalence of gastrointestinal symptoms among patients with A-ATTRv compared to carriers (76.47% vs. 10%, *p* = 0.0001), as shown in Table 1.

### Presence of SIBO in carriers and patients with A-ATTRv

The overall prevalence of SIBO in the study population was 62.2%, with 23 out of 37 individuals affected. Among asymptomatic carriers, 12 out of 20 were diagnosed with SIBO, resulting in a prevalence of 60%. In patients with A-ATTRv, 11 out of 17 had SIBO, corresponding to a prevalence of 64.7%. No significant difference was observed between the two groups, with a *p*-value of 1.000, as shown in Table 2.

**Table 2:** Analysis of SIBO results in the study population

SIBO Result		Total (*n* = 37)	Carriers (*n* = 20)	ATTRv Patients (*n* = 17)
Positive	Number	23	12	11
	(%)	62,16%	60%	64,70%
Negative	Number	14	8	6
	(%)	37,84%	40%	35,30%

SIBO Subtype		Total (n = 23)	Carriers (n = 12)	ATTRv Patients (n = 11)
H_2_ SIBO	Number	2	0	2
	(%)	8,69%	0%	18,18%
	95% CI	(2.42–26.80)	(0.00, 24.25)	(5.14. 47.70)
CH_4_ SIBO	Number	13	7	6
	(%)	56,52%	58,53%	54,54%
	95% CI	(36.81–74.37)	(31.95–80.67)	(28.01–78.73)
Both subtypes	Number	8	5	3
	(%)	34,79%	41,47%	27,28%
	95% CI	(18.81–55.11)	(19.33–68.05)	(9.75–56.56)

### Frequency of different subtypes of SIBO in carriers and patients with A-ATTRv

The frequency of various SIBO subtypes is summarized in Table 2. Statistical analyses indicated no significant relationship between the frequency of different SIBO subtypes in asymptomatic carriers and patients with A-ATTRv (*p* = 0.2811). Additionally, comparisons between H_2_ (*p* = 0.2174) and CH_4_ SIBO (*p* = 1.0000) individually revealed no significant differences between carriers and patients. The distribution of single subtype versus combined subtypes also showed no statistically significant differences between the groups (*p* = 0.6668). The 95% confidence intervals provided in Table 2 further support these non-significant differences.

### Digestive symptoms in individuals with SIBO

Table 3 shows the presence of digestive symptoms in individuals who tested either positive or negative for SIBO. We observed that there is no significant relationship between the presence of SIBO and the different digestive disturbances evaluated (*p* = 0.7332). We also analysed the presence of different digestive symptoms in the different SIBO subtypes. No statistically significant differences were detected in individuals with CH_4_ SIBO who presented alternating episodes of diarrhea and constipation (*p* = 0.6600) compared to the other two types (H_2_ and combination). No relationship was observed between individuals with different SIBO subtypes and the GI symptoms studied.

**Table 3:** Patients with SIBO and digestive symptoms

		Digestive symptoms	No symptoms
SIBO + (*n* = 23)	Number	9	14
	(%)	39,13%	60,87%
SIBO– (*n* = 14)	Number	7	7
	(%)	50%	50%

## Discussion

Our study observed a notably higher prevalence of SIBO in both carriers and symptomatic patients with A-ATTRv compared to general population estimates [23, 24]. However, the lack of a control group comprising individuals without the ATTR gene variant limits our ability to establish causality between ATTRv amyloidosis and SIBO. The observed prevalence, more than two to three times higher than typical rates in the general population, highlights the need for further exploration of gastrointestinal symptoms in A-ATTRv. Future research, including larger cohorts with appropriate control groups, is crucial to determine whether the increased prevalence of SIBO in these patients is directly related to the disease or influenced by other underlying mechanisms.

According to our results, SIBO prevalence did not significantly differ between A-ATTRv patients and asymptomatic carriers, despite the higher frequency of GI manifestations in the symptomatic group. This suggests that while bacterial overgrowth may be present, it is unlikely to be the sole or primary cause of gastrointestinal symptoms in these patients. This observation suggests that GI symptoms in ATTRv amyloidosis may be multifactorial and not exclusively attributable to SIBO.

Patients with A-ATTRv may experience a variety of GI symptoms. Bacterial overgrowth can be due to dysmotility and gastroparesis, which alter gastric emptying and intestinal motility, creating an environment for microbial growth [21]. Some studies, although with limited patient numbers, have suggested that gastric retention associated with A-ATTRv may lead to SIBO and impaired nutrient absorption [20]. Additionally, American Guidelines describe amyloidosis as a potential risk factor for SIBO, although there are no studies specifically confirming this in A-ATTRv [39].

The heterogeneity of the cohort in this study reflects the diverse clinical manifestations of variant transthyretin amyloidosis (ATTRv) observed in the endemic focus of Mallorca. While ATTRv is traditionally characterized by a predominant neurological phenotype, particularly in early-onset cases, the disease can also present with significant cardiac involvement or mixed phenotypes. This variability may influence the gastrointestinal symptoms reported, with potential contributions from autonomic dysfunction and systemic disease burden. For example, autonomic neuropathy can lead to delayed gastric emptying and impaired peristalsis, creating conditions that favor bacterial overgrowth and dysbiosis. The inclusion of patients with exclusively cardiac complications is supported by the multisystemic nature of ATTRv, as demonstrated in the EMPATIa study, which reported gastrointestinal (GI) symptoms in nearly half of affected individuals, including those with cardiomyopathy [41]. Additionally, findings by González-Moreno et al. [42] and Zampino et al. [43] reinforce that autonomic dysfunction contributes to GI impairment, even in patients with primary cardiac manifestations.

In our study, we show the presence of a high prevalence of SIBO in both carriers and patients with A-ATTRv, as evidenced by the breath test. However, the lack of significant differences between groups suggests that the presence of SIBO may not be the primary driver of GI symptoms in ATTRv amyloidosis. Instead, it is likely that multiple overlapping factors—such as altered gut motility, autonomic dysfunction, and systemic inflammation—contribute to gastrointestinal disturbances in these patients. Moreover, patients exhibited a higher frequency of digestive symptoms compared to asymptomatic carriers, consistent with previous findings [18].

Among intestinal microbiota dysbiosis, H_2_ and CH_4_ SIBO are the most studied conditions. However, diagnostic variability remains a major challenge. While guidelines from the American Gastroenterological Association and the North American Consensus provide standardized recommendations, considerable discrepancies exist in breath test interpretation, substrate selection, and diagnostic cut-offs, impacting the reliability of SIBO detection. A key challenge is the absence of a universally accepted gold standard for SIBO diagnosis, which contributes to inconsistencies across studies. Additionally, confounding factors such as inadequate pre-test preparation or differences in intestinal transit times could influence results. Some criteria also consider a rise of ≥ 12 ppm from baseline within 90 min as diagnostic. Given these factors, the diagnostic thresholds used in our study may have influenced the results, particularly in the reported prevalence of SIBO and IMO. This highlights the need for further research to refine diagnostic criteria and improve the accuracy of breath testing for these conditions [39, 40].

It is important to consider that other gut dysbiosis conditions, such as small intestinal fungal overgrowth (SIFO), were not assessed in this study, despite their potential role in gastrointestinal symptoms [31]. Fungal overgrowth, alterations in bile acid metabolism, and changes in gut permeability could all contribute to the gastrointestinal symptoms observed in A-ATTRv. Once standardized diagnostic criteria for SIFO are established, future studies should investigate its prevalence in A-ATTRv patients to provide a more comprehensive understanding of gut microbiota alterations in this disease.

In our study, lactitol was chosen as the substrate due to its local availability and the standardized protocols at our institution. However, it is important to acknowledge that lactulose and lactitol can accelerate gastrointestinal transit, potentially leading to false-negative results when compared to glucose. While glucose is rapidly absorbed in the proximal small intestine, leading to a higher specificity for detecting proximal SIBO, lactulose and lactitol travel further along the gastrointestinal tract, which may result in a delayed or absent peak in hydrogen production. This phenomenon is particularly relevant in the context of breath testing for SIBO, where the American Gastroenterological Association notes that the timing of the first peak in hydrogen production is critical for accurate diagnosis [39].

Currently, a new technology based on capsules is being developed to provide real-time measurements for SIBO. Intraluminal gases such as H_2_, carbon dioxide (CO_2_), and CH_4_ are measured as the capsule travels through the GI tract using sensors and measuring conductivity. Ultrasound can be used as an accurate marker for capsule location. A study concluded that the capsule had high sensitivity for measuring intraluminal H_2_ concentrations, providing information on the site of intestinal gas production, demonstrating great safety and reliability [27, 38].

We found no direct correlation between gender and SIBO. However, the literature suggests that SIBO may be more common in women [39], probably due to differences in GI tract anatomy and physiology between genders, as well as hormonal factors that could influence intestinal motility and microbiota [39]. Additionally, it can be associated with conditions such as irritable bowel syndrome and pelvic inflammatory disease, which are more common in women. Further research is needed to understand any association between gender and SIBO.

The relationship between A-ATTRv and SIBO has been under investigation during the last years. It is important to note that GI symptoms can vary widely among A-ATTRv patients and may be caused by different factors besides SIBO. Other potential contributors include chronic inflammation, metabolic alterations, gut microbiome imbalances, and polyneuropathy-associated dysautonomia. There is weak evidence suggesting that SIBO could be a complication associated with A-ATTRv [20] and we failed to prove it.

However, we are aware that our study has several limitations:

- Sample Size: A major limitation of our study is the small sample size, which likely reduced statistical power (6%) and may have limited our ability to detect subtle but potentially meaningful differences in SIBO prevalence between ATTRV30M carriers and patients. While no significant differences were observed, this does not rule out the possibility that a larger study might identify an association. Additionally, the limited recruitment period may have affected the generalizability of our findings, potentially missing seasonal or other population-based variations.
- Common Contributing Factors: Both patients and asymptomatic carriers may share common factors that contribute to SIBO development, such as autonomic dysfunction, altered intestinal motility, or microbiota changes, complicating the interpretation of results.

Test Sensitivity: Another key limitation is the sensitivity of the breath tests used for SIBO diagnosis, which may have led to false negatives and an underestimation of true SIBO prevalence. The lactitol breath test does not capture all SIBO subtypes, and we did not assess other forms of microbial dysbiosis, such as small intestinal fungal overgrowth (SIFO), which can present with similar symptoms. These factors may have influenced our ability to detect a genuine relationship between A-ATTRv and SIBO, warranting further investigation in studies using more comprehensive diagnostic approaches.

While these factors may limit the generalizability of our findings, the exploratory nature of the study provides valuable initial insights into the potential lack of association between A-ATTRv amyloidosis and SIBO. Given the study’s limitations, future research should prioritize several key areas to strengthen and confirm these findings. First, increasing the sample size with larger cohorts and extending the recruitment period would provide more robust data, enhancing statistical power and improving the generalizability of results. This would be the most impactful step in validating our findings. Second, employing more sensitive diagnostic tools, such as real-time intraluminal gas measurement capsules, which offer greater accuracy in diagnosing SIBO, would refine the assessment of bacterial overgrowth and enhance the precision of future studies on SIBO in A-ATTRv patients. Lastly, investigating other forms of gut dysbiosis, such as small intestinal fungal overgrowth (SIFO), could provide a more comprehensive understanding of gastrointestinal symptoms in this patient population and further clarify the complex relationship between gut microbiota disturbances and A-ATTRv amyloidosis.

## Conclusions

Patients with A-ATTRv did not show a higher prevalence of any SIBO subtype compared to asymptomatic carriers, suggesting that gastrointestinal symptoms are not mainly due to SIBO. These symptoms are likely linked to factors such as autonomic dysfunction, impaired gut motility, and mucosal changes. However, it’s unclear whether SIBO occurs independently of the disease stage or if subclinical ATTR amyloidosis in asymptomatic patients could influence SIBO prevalence. SIBO should still be considered a potential contributing factor in A-ATTRv patients with gastrointestinal symptoms, even though this study did not find a significant association. Further research is needed to better understand the relationship between SIBO and ATTR amyloidosis, particularly in the early stages of the disease.

## Data Availability

The data are available upon reasonable request. All data relevant to the study are included in the article.
